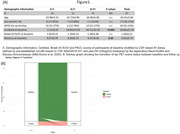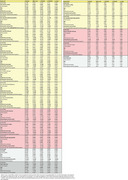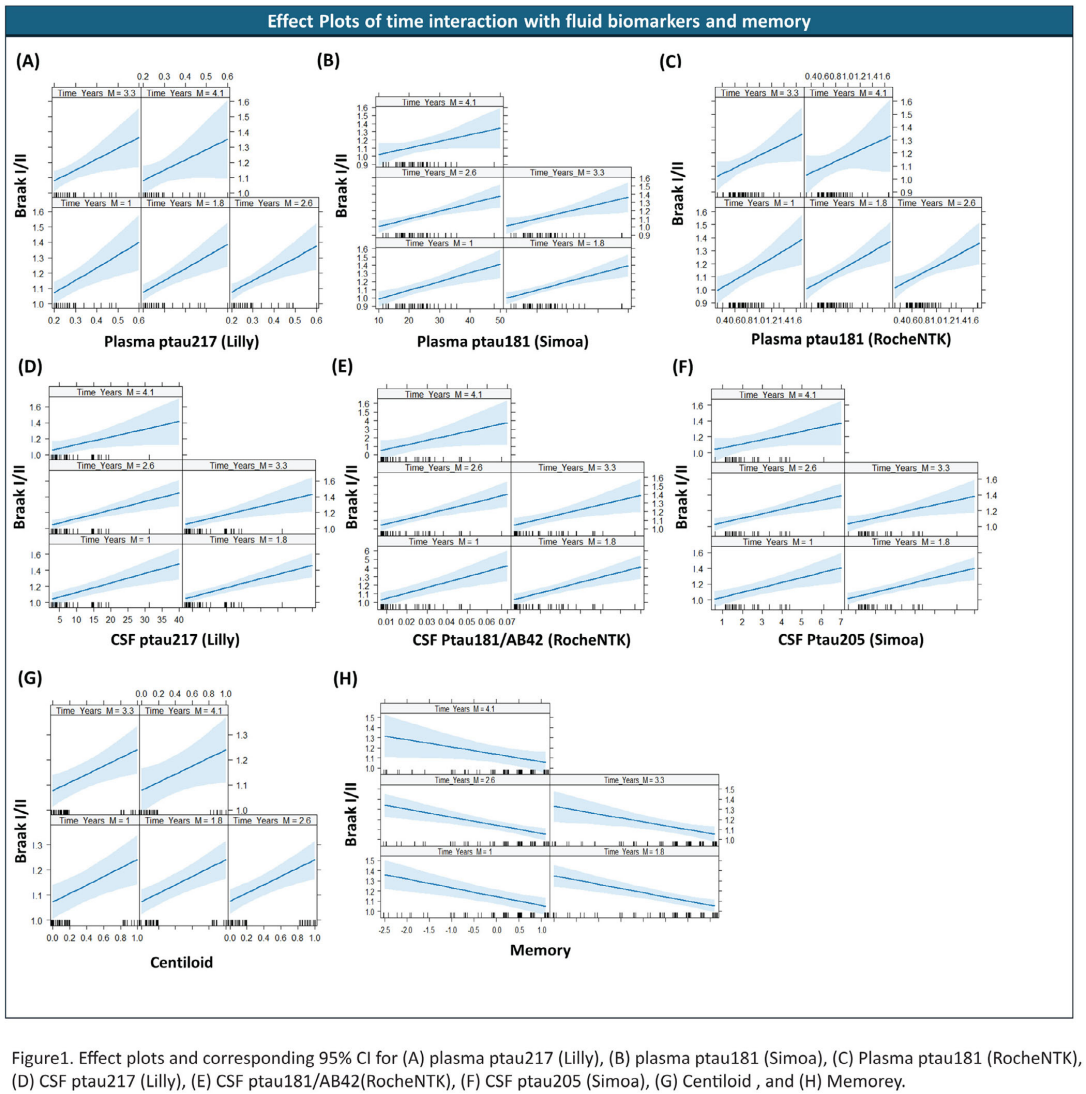# Exploring Associations Between Longitudinal [^18^F]RO‐948 Tau PET, Baseline Fluid tau Biomarkers, and memory in the Early Alzheimer's Disease *continuum*

**DOI:** 10.1002/alz70856_106562

**Published:** 2026-01-07

**Authors:** Mahnaz Shekari, Armand González Escalante, David López‐Martos, Marta Milà‐Alomà, Gonzalo Sánchez‐Benavides, Anna Brugulat‐Serrat, Aida Niñerola‐Baizán, Carles Falcon, Nicholas J. Ashton, Thomas Karikari, Juan Lantero Rodriguez, Anniina Snellman, Theresa A. Day, Jeffrey L. Dage, Paula Ortiz‐Romero, Matteo Tonietto, Gregory Klein, Gwendlyn Kollmorgen, Clara Quijano‐Rubio, Eugeen Vanmechelen, Carolina Minguillón, Karine Fauria, Andrés Perissinotti, Jose Luis Molinuevo, Henrik Zetterberg, Kaj Blennow, Gemma Salvadó, Oriol Grau‐Rivera, David Vállez‐Garcia, Marc Suárez‐Calvet, Juan Domingo Gispert, ALFA+ study

**Affiliations:** ^1^ Barcelonaβeta Brain Research Center (BBRC), Pasqual Maragall Foundation, Barcelona, Spain; ^2^ Hospital del Mar Research Institute (IMIM), Barcelona, Spain; ^3^ Universitat Pompeu Fabra, Barcelona, Spain; ^4^ Department of Radiology and Biomedical Imaging, University of California, San Francisco, San Francisco, CA, USA; ^5^ Centro de Investigación Biomédica en Red de Fragilidad y Envejecimiento Saludable (CIBERFES), Instituto de Salud Carlos III, Madrid, Spain; ^6^ Centro de Investigación Biomédica en Red de Fragilidad y Envejecimiento Saludable (CIBERFES), Instituto de Salud Carlos III, Barcelona, Spain; ^7^ Hospital del Mar Research Institute, Barcelona, Spain; ^8^ Global Brain Health Institute, San Francisco, CA, USA; ^9^ Nuclear Medicine Department, Hospital Clínic Barcelona, Barcelona, Spain; ^10^ Centro de Investigación Biomédica en Red de Bioingeniería, Biomateriales y Nanomedicina (CIBER‐BBN), Madrid, Spain; ^11^ Banner Alzheimer's Institute and University of Arizona, Phoenix, AZ, USA; ^12^ King's College London, Institute of Psychiatry, Psychology & Neuroscience, Maurice Wohl Clinical Neuroscience Institute, London, United Kingdom; ^13^ Department of Psychiatry and Neurochemistry, Institute of Neuroscience & Physiology, the Sahlgrenska Academy at the University of Gothenburg, Mölndal, Sweden; ^14^ NIHR Biomedical Research Centre for Mental Health and Biomedical Research Unit for Dementia at South London and Maudsley, NHS Foundation, London, United Kingdom; ^15^ Wallenberg Centre for Molecular and Translational Medicine, University of Gothenburg, Gothenburg, Sweden; ^16^ University of Pittsburgh, Pittsburgh, PA, USA; ^17^ Turku PET Centre, University of Turku, Turku, Finland; ^18^ Department of Psychiatry and Neurochemistry, Institute of Neuroscience & Physiology, the Sahlgrenska Academy at the University of Gothenburg, Mölndal, Sweden; ^19^ Lilly Research Laboratories, Eli Lilly and Company, Indianapolis, IN, USA; ^20^ Stark Neurosciences Research Institute, Indiana University School of Medicine, Indianapolis, IN, USA; ^21^ Roche Pharma Research and Early Development, FHoffmann‐La RocheLtd, Basel, Switzerland; ^22^ Roche Diagnostics GmbH, Penzberg, Germany; ^23^ Roche Diagnostics International Ltd., Rotkreuz, Switzerland; ^24^ ADx NeuroSciences NV, Technologiepark 94, Gent, Belgium; ^25^ Lundbeck A/S, Copenhagen, Denmark; ^26^ Clinical Neurochemistry Laboratory, Sahlgrenska University Hospital, Mölndal, Västra Götalands län, Sweden; ^27^ Department of Neurodegenerative Disease, UCL Institute of Neurology, London, United Kingdom; ^28^ UK Dementia Research Institute, University College London, London, United Kingdom; ^29^ Department of Psychiatry and Neurochemistry, Institute of Neuroscience and Physiology, The Sahlgrenska Academy, University of Gothenburg, Mölndal, Sweden; ^30^ Clinical Neurochemistry Laboratory, Sahlgrenska University Hospital, Mölndal, Sweden; ^31^ Clinical Memory Research Unit, Department of Clinical Sciences Malmö, Lund University, Lund, Sweden; ^32^ Servei de Neurologia, Hospital del Mar, Barcelona, Spain; ^33^ Hospital del Mar Research Institute, Barcelona, Barcelona, Spain; ^34^ AstraZeneca, Barcelona, Spain

## Abstract

**Background:**

The relationship between longitudinal tau‐PET trajectories and fluid tau biomarkers in early Braak stages is crucial for understanding Alzheimer's disease (AD) progression and identifying predictive biomarkers for early diagnosis and intervention. This study examines how baseline plasma and cerebrospinal fluid (CSF) tau biomarkers, amyloid plaques, and cognitive performance interact with longitudinal tau‐PET trajectories in cognitively unimpaired (CU) individuals.

**Method:**

Forty‐six CU individuals from ALFA+ cohort were included, each with two [18F]RO‐948 tau‐PET scans (∆t=2.31±0.34 years), two T1‐weighted MRIs, and baseline [18F]flutemetamol amyloid‐PET (Figure 1). Baseline fluid tau biomarkers, measured using RocheNTK, RocheElecsys, Simoa, and Lilly assays, as well as memory, were also available. [18F]RO‐948 uptake was measured in entorhinal (BraakI/II), limbic (BraakIII/IV), and neocortical (BraakV/VI) regions and normalized to the inferior cerebellum to render SUVR. Amyloid‐PET was quantified using Centiloid. Tau‐PET stage transitions from baseline to follow‐up assessed using predefined positivity thresholds. Linear mixed‐effects models evaluated associations between baseline biomarkers, Centiloid, memory, and tau‐PET SUVR over time, adjusting for age, sex, APOE‐ε4, and time intervals.

**Result:**

Three participants (6.52%) were positive for BraakI/II at both baseline and follow‐up (Stable‐Positive), two (4.34%) transitioned to positive (Progressors), while the majority (41, 89.13%) remained negative (Stable‐Negative) (Figure 1). Interaction between baseline predictors and time was not statistically significant for any predictive variables (Table 2). However, higher plasma ptau181 and ptau217 levels were significantly associated with higher baseline BraakI/II SUVR, while only plasma ptau181 showed a significant association with baseline BraakIII/IV. For CSF, higher ptau217 and ptau181/Aβ42 ratio were significantly associated with higher baseline tau‐PET SUVR in both BraakI/II and BraakIII/IV (Table 1 & Figure 2). Additionally, Centiloid was positively associated with tau‐PET SUVR in BraakI/II but not in other regions. Lower baseline memory scores were significantly associated with higher tau‐PET SUVR in BraakI/II and BraakIII/IV in baseline.

**Conclusion:**

Our findings support the potential of plasma and CSF tau biomarkers as early indicators of Alzheimer's pathology in cognitively unimpaired individuals. The negative association between memory and tau‐PET SUVR suggests subtle cognitive differences may reflect underlying tau accumulation. However, no significant longitudinal effects were observed, likely due to the limited sample size or short follow‐up.